# Breathing variability—implications for anaesthesiology and intensive care

**DOI:** 10.1186/s13054-021-03716-0

**Published:** 2021-08-05

**Authors:** Oscar F. C. van den Bosch, Ricardo Alvarez-Jimenez, Harm-Jan de Grooth, Armand R. J. Girbes, Stephan A. Loer

**Affiliations:** Departments of Anesthesiology and Intensive Care, Amsterdam UMC, VUMC, ZH 6F 003, De Boelelaan 1117, 1081 HV Amsterdam, The Netherlands

**Keywords:** Respiratory variability, Control of breathing, Spontaneous respiration, Coefficient of variation, Detrended fluctuation analysis, Entropy analysis, Perioperative period, Spontaneous breathing trial

## Abstract

The respiratory system reacts instantaneously to intrinsic and extrinsic inputs. This adaptability results in significant fluctuations in breathing parameters, such as respiratory rate, tidal volume, and inspiratory flow profiles. Breathing variability is influenced by several conditions, including sleep, various pulmonary diseases, hypoxia, and anxiety disorders. Recent studies have suggested that weaning failure during mechanical ventilation may be predicted by low respiratory variability. This review describes methods for quantifying breathing variability, summarises the conditions and comorbidities that affect breathing variability, and discusses the potential implications of breathing variability for anaesthesia and intensive care.

## Background

**Fig. 1:**
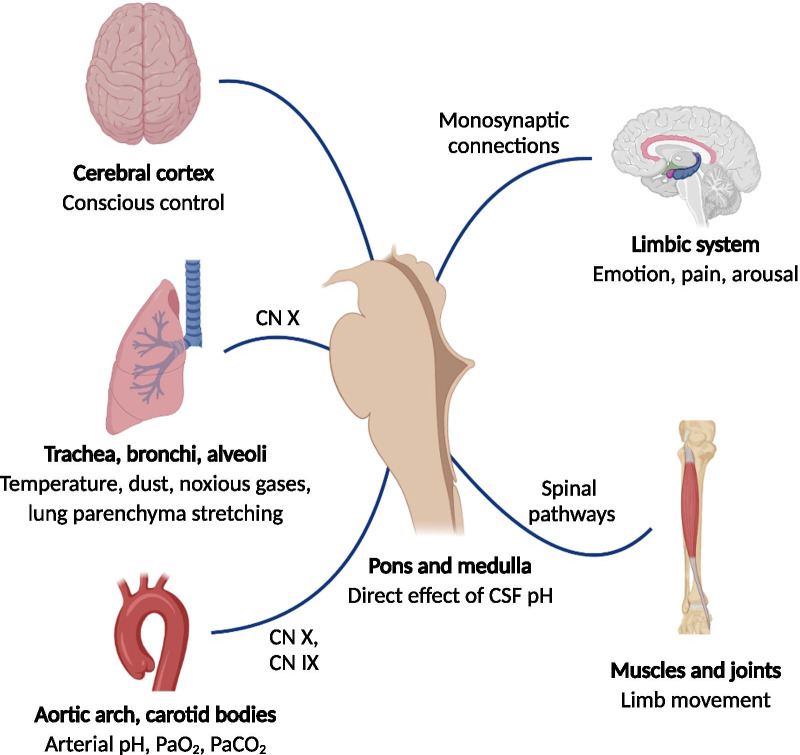
Input to the respiratory centre in the medulla oblongata and the pons

The control of breathing involves a complex system that balances the opposing goals of efficiency, redundancy, responsiveness, and stability [1]. It is characterised by myriad inputs, internal pacemakers, positive and negative feedback loops, and nonlinear interactions between different components (Fig. 1). This results in fluctuations in breathing parameters, including the respiratory rate, tidal volume, and airflow profiles (Table 1).

**Table 1:** Tidal breathing parameters

RR	Respiratory rate
*T*_I_	Inspiratory time
*T*_E_	Expiratory time
*T*_tot_	Total breath cycle time
*T*_i_/*T*_tot_	Ratio inspiratory time: total respiratory time
*V*_T_	Tidal volume
*V*_T_/*T*_i_	Mean inspiratory flow
MV	Minute ventilation
*V*_E CO2_	Expired CO_2_ volume per breath
*F*_E CO2_	Mixed expired CO_2_ fraction per breath
*F*_et CO2_	End-expiratory CO_2_ fraction

If this regulation is too rigid, respiratory variability is low or absent, and the respiratory system cannot adequately react to stimuli. In contrast, if the respiratory system is overreacting to internal and external stimuli, the system shows large fluctuations and loses control.

The exact determinants of breathing variability are not precisely known; however, it has been shown that the increase and decrease in breathing variability are strongly associated with pathological states. Normal breathing variability is influenced by several factors, such as aging, cognitive load, sleep pattern, and hypoxia, as well as medical conditions such as anxiety, obstructive or restrictive lung disease, and arterial hypertension. During anaesthesia and intensive care, additional factors, such as drugs and the effects of mechanical ventilation, may also influence breathing variability.

This review describes the methods for quantifying breathing variability, summarises the conditions and comorbidities that affect breathing variability, and discusses the potential implications of breathing variability for anaesthesia and intensive care.

### Normal respiratory variability

The regulation of breathing facilitates adequate gas exchange for metabolic needs. The impulses for inspiration and expiration are generated within the respiratory centre in the medulla oblongata after receiving and processing input from various subsystems (Fig. 1 and Table 2). Consequently, respiration is characterised by constant fluctuations in rate, rhythm, depth, and duration [2, 3]. For instance, this physiological variability may range between 19 and 34% for tidal volume and between 16 and 22% for respiratory rate, expressed as the coefficient of variation, in awake persons (Table 3). The respiratory system receives input mainly from central and peripheral chemoreceptors, mechanoreceptors within the airways and alveoli, locomotion receptors of muscles and joints, and the (para)limbic system. The breathing pattern may be influenced to an extent and voluntarily controlled, shortly, via input from the cerebral cortex. For this purpose, the forebrain sends signals to the respiratory centre via independent pathways, overruling other inputs. Further factors include afferent input from the vagus nerve and its branches, such as the superior laryngeal nerves. External vagal stimulation has been shown to decrease respiratory rate [4], while stimulation of the superior laryngeal nerves may affect the chest wall and airway muscles [5]. Inspiratory time and tidal volume remain strongly correlated, suggesting a constant flow at a steady chemical drive [6, 7].

**Table 2:** Control of spontaneous respiration

	Location	Stimulus	Pathway
Central chemoreceptors	Ventral medulla	CSF pH	Direct effect
Peripheral chemoreceptors	Bifurcation of common carotid artery, aortic arch	Arterial pH, PaO_2_, PaCO_2_,	Glossopharyngeal nerve, vagal nerve
Mechanoreceptors	Tracheal and bronchial muscle spindles	Stretching of lung parenchyma	Vagal nerve
	Airways and alveoli	Temperature, dust, noxious gases	Vagal nerve
Locomotion receptors	Muscles and joints	Limb movement	Spinal pathways
Other	Cerebral cortex and (para)limbic system	Emotion, pain, arousal	Monosynaptic connections
	Cerebral cortex	Conscious/voluntary control	Corticospinal pathways

**Table 3:** Conditions affecting respiratory variability

Factor	Reference	Respiratory measurements	Measure of variability	
Age	Tobin et al. [26]	Inductive plethysmography	CV	Young versus old subjects: RR: 0.16 versus 0.17 TV: 0.22 versus 0.28* MV: 0.22 versus 0.27* IT: 0.19 versus 0.21
	Peng et al. [11]	Inductive plethysmography	DFA (α)	Healthy young versus healthy elderly subjects (male): BT: 0.68 ± 0.07 versus 0.60 ± 0.08* Healthy young versus healthy elderly subjects (female): BT: 0.70 ± 0.07 versus 0.67 ± 0.06
Sleep	Hudgel et al. [16]	Nasal CPAP mask, pneumotachography	CV	Awake versus NREM sleep (elderly): TV: 0.24 ± 0.04 versus 0.25 ± 0.04 MV: 0.20 ± 0.02 versus 0.25 ± 0.05 IT: 0.15 ± 0.02 versus 0.11 ± 0.01 BT: 0.15 ± 0.02 versus 0.10 ± 0.05* Awake versus NREM sleep (young): TV: 0.23 ± 0.04 versus 0.11 ± 0.02* MV: 0.14 ± 0.02 versus 0.10 ± 0.11 IT: 0.17 ± 0.02 versus 0.08 ± 0.01* BT: 0.16 ± 0.02 versus 0.08 ± 0.01*
	Rostig et al. [17]	Full-face mask, pneumotachography	CV	NREM versus REM sleep: TV: 0.11 ± 0.01 versus 0.28 ± 0.08* MV: 0.11 ± 0.03 versus 0.21 ± 0.09* RR: 0.09 ± 0.03 versus 0.20 ± 0.04* IT: 0.10 ± 0.02 versus 0.15 ± 0.03
			DFA, STC (α_1_)	TV: 0.89 ± 0.09 versus 1.18 ± 0.14 MV: 0.96 ± 0.10 versus 1.18 ± 0.16 RR: 0.79 ± 0.06 versus 0.95 ± 0.14 IT: 0.68 ± 0.04 versus 0.76 ± 0.10
			DFA, LTC (α_2_)	TV: 0.51 ± 0.09 versus 0.81 ± 0.15 MV: 0.51 ± 0.09 versus 0.77 ± 0.13 RR: 0.57 ± 0.05 versus 0.85 ± 0.12 IT: 0.55 ± 0.07 versus 0.76 ± 0.11
Hypertension	Anderson et al. [30]	Inductive plethysmography	RMSSD	Lower versus upper tertile of blood pressure: TV: 147 ± 38 versus 219 ± 22* MV: 1.84 ± 0.32 versus 1.91 ± 0.46* RR: 3.56 ± 0.50 versus 4.72 ± 0.73*
Children with anxiety disorder	Pine et al. [32]	Spirometry and respiratory canopy	SD	Anxiety disorder versus normal controls: TV: 98 ± 101 versus 39 ± 27* MV: 2.0 ± 2.0 versus 0.8 ± 0.4* RR: data not reported, P = 0.06
Panic disorder	Martinez et al. [34]	Spirometry and respiratory canopy	SD	Panic disorder versus normal controls: TV: 191 ± 184 versus 84 ± 43* MV: 2.36 ± 1.80 versus 1.37 ± 0.68* RR: 4.59 ± 2.75 versus 3.58 ± 2.43
			MSSD	TV: 308 ± 358 versus 144 ± 152* MV: 4.27 ± 5.05 versus 2.12 ± 1.84* RR: 6.90 ± 7.75 versus 5.38 ± 5.13
	Yeragani et al. [36]	Inductance plethysmography	CV	Panic disorder versus normal controls: TV: 0.54 ± 0.22 versus 0.33 ± 0.15 (standing)* RR: 0.39 ± 0.17 versus 0.45 ± 0.10 TV: 0.23 ± 0.10 versus 0.32 ± 0.27 (supine) RR: 0.29 ± 0.12 versus 0.32 ± 0.11
			LLE	Panic disorder versus normal controls: 0.10 ± 0.01 versus 0.086 ± 0.02 (standing)* 0.09 ± 0.02 versus 0.09 ± 0.02 (supine)
			ApEn	Panic disorder versus normal controls: 0.40 ± 0.13 versus 0.27 ± 0.12 (standing)* 0.30 ± 0.0 versus 0.29 ± 0.12 (supine)
Cognitive load	Vlemincx et al. [21]	Inductance plethysmography	CV	Complex arithmetic task versus baseline: TV: 0.36 ± 0.16 versus 0.24 ± 0.14* MV: 0.26 ± 0.08 versus 0.20 ± 0.08* RR: 0.18 ± 0.08 versus 0.16 ± 0.07
			AR	Complex arithmetic task versus baseline: TV: 0.11 ± 0.23 versus 0.20 ± 0.18* MV: 0.30 ± 0.24 versus 0.28 ± 0.18 RR: 0.12 ± 0.15 versus 0.26 ± 0.20*
	Grassmann et al. [22]	Nasal capnometry	CV	High demanding mental multi-task versus baseline: RR: 0.13 ± 0.05 versus 0.19 ± 0.09*
			AR	High demanding mental multi-task versus baseline: RR: 0.05 ± 0.11 versus 0.13 ± 0.18*
COPD	Loveridge et al. [37]	Inductance plethysmography	CV	COPD versus normal controls: TV 0.253 versus 0.337* MV 0.221 versus 0.280* RR 0.170 versus 0.220 IT 0.178 versus 0.229*
Asthma	Hmeidi et al. [38]	Structured light plethysmography	IQR	Asthma (prebronchodilator) versus normal controls: RR: 3.93 (2.57) versus 3.32 (2.2) IE50: 0.63 (0.32) versus 0.47 (0.18)* Asthma (prebronchodilator) versus asthma (postbronchodilator): RR 3.93 (2.57) versus 4.62 (2.34) IE50: 0.63 (0.32) versus 0.60 (0.38)*
Asthma	Seppa et al. [39]	Impedance pneumography	CSR_min_	High-risk group versus low-risk group: Flow-volume curve 0.995 [0.984–0.999] versus 0.998 [0.994–0.999]*
			NL_min_	High-risk group versus low-risk group: Flow signal 14.3 [0.00–48.7] versus 30.3 [0.00–42.7]*
Restrictive lung disease	Brack et al. [42]	Inductance plethysmography	CV	Restrictive lung disease versus normal controls: TV 0.22 ± 0.05 versus 0.50 ± 0.20* IT 0.22 ± 0.05 versus 0.33 ± 0.12* ET 0.22 ± 0.07 versus 0.41 ± 0.19* MV 0.24 ± 0.06 versus 0.42 ± 0.16*
			AR	TV 0.43 ± 0.14 versus 0.23 ± 0.12* IT 0.25 ± 0.17 versus 0.21 ± 0.14 ET 0.28 ± 0.13 versus 0.12 ± 0.15* MV 0.39 ± 0.16 versus 0.27 ± 0.14
Lung dysmaturity in infancy	Fouzas et al. [43]	Full face mask, flowmeter	CV	Preterm non-CLDI infants versus term infants: TV 0.09 ± 0.03 versus 0.09 ± 0.02 Preterm moderate/severe CLDI versus term infants: TV 0.07 ± 0.02 versus 0.09 ± 0.02*
			2D dispersion, Poincaré	Preterm non-CLDI infants versus term infants: TV 147 ± 82.2 versus 143 ± 64.3 Preterm moderate/severe CLDI versus term infants: TV 58.9 ± 40.7 versus 143 ± 64.3*
			3D dispersion, Poincaré	Preterm non-CLDI infants versus term infants: TV 1156 ± 906 versus 1073 ± 670 Preterm moderate/severe CLDI versus term infants: TV 284 ± 253 versus 1073 ± 670*
	Usemann et al. [44]	Full face mask, flowmeter	CV	Preterm versus term infants: *R*_int_ 20.2 ± 8.4 versus 29.6 ± 14.9*
After major abdominal surgery	Van den Bosch et al. [56]	Impedance pneumography	CV	RR versus TV: 0.21 ± 0.06 versus 0.37 ± 0.12* TV versus MV: 0.37 ± 0.12 versus 0.41 ± 0.12*
Organ dysfunction syndrome in ICU	Bradley et al. [63]	Capnography	CV	Before versus after sedation interruption, low MODS: RR 0.17 ± 0.08 versus 0.28 ± 0.16* Before versus after sedation interruption, high MODS: RR 0.23 ± 0.12 versus 0.20 ± 0.12
			RMSSD	Before versus after sedation interruption, low MODS: RR 0.86 ± 0.53 versus 1.7 ± 1.3 Before versus after sedation interruption, high MODS: RR 1.3 ± 0.79 versus 1.0 ± 0.79*
			ApEn	Before versus after sedation interruption, low MODS: RR 0.48 ± 0.16 versus 0.46 ± 0.15 Before versus after sedation interruption, high MODS: RR 0.42 ± 0.16 versus 0.49 ± 0.17*
			DFA, STC (α_1_)	Before versus after sedation interruption, low MODS: RR 0.64 ± 0.19 versus 0.70 ± 0.15 Before versus after sedation interruption, high MODS: RR 0.69 ± 0.09 versus 0.68 ± 0.17
			DFA, LTC (α_2_)	Before versus after sedation interruption, low MODS: RR 1.17 ± 0.78 versus 0.33 ± 0.41 Before versus after sedation interruption, high MODS: RR 0.25 ± 0.22 versus 0.40 ± 0.35*

CV coefficient of variation, TV tidal volume, MV minute ventilation, IT inspiratory time, BT breath time, RR respiratory rate, DFA detrended fluctuation analysis, STC short-term correlations, LTC long-term correlations, (R)MSSD (root) mean square of successive differences, SD standard deviation, LLE largest Lyapunov exponent (a measure of chaos), ApEn approximate entropy (a measure of regularity), AR autocorrelation at one breath lag, COPD chronic obstructive pulmonary disease, IE50 thoracoabdominal displacement rate at 50% of inspiratory displacement divided by thoracoabdominal expiratory displacement date at 50% of expiratory displacement, CSR_min_ minimum curve shape correlation, NLmin minimum noise limit, ET expiratory time, CLDI chronic lung disease of infancy *R*_int_ airway resistance with interrupter technique, MODS multiple organ dysfunction syndrome* *P* < 0.05

Different models have been developed to predict respiratory variability. One of the first models used a semi-mechanistic approach based on feedback loops of measured (cardiac output and mixed venous blood CO_2_ partial pressure) and estimated parameters (CO_2_ sensitivity, mean lung volume for CO_2_, circulating time) and integrated the result into a compartmental model using differential Eqs. [8]. For these calculations, several assumptions were necessary, including constant hemodynamic parameters, absence of intracardiac or pulmonary shunting, and instant intra-alveolar equilibration of CO_2_ and oxygen tensions. Another more recent approach used spectral analysis of all variables to predict oscillatory rhythmicity [9]. This model incorporated inspiratory and expiratory times and volumes, as well as end-tidal CO_2_ partial pressures and driving parameters. Of note, both models were based on measurements of healthy participants and can be extrapolated to patients with caution.

### Quantification of respiratory variability

Different methods are available for the quantification of breathing variability, including quantitative time series analysis, detrended fluctuation analysis, entropy analysis, frequency distribution analysis, spectrum analysis, and power-law analysis [1]. In this section, we focus on the first three techniques.

#### Quantitative time series analysis

A quantitative time series analysis evaluates the standard deviation or interquartile range over time, such as the standard deviation of tidal volume or respiratory rate. The coefficient of variation (CV), defined as the ratio of the standard deviation to the mean, shows the extent of variability in relation to the mean of the data series (Eq. 1). The coefficient of variation is useful for comparing datasets with different units or means. It can be used as a measure of short- and long-term variations, depending on the subsets of data analysed. The extent of variability between successive breaths was calculated as the root mean square of successive differences (RMSSD) over consecutive breaths (short-term variability, Eq. 2). A quantitative time series analysis shows the overall degree of variability or “*quantitative variability*”.

1 $$\begin{array}{*{20}c} { {\text{CV}}\left( x \right) = \frac{{\sqrt {\frac{1}{N}\sum \left( {x - \overline{x}} \right)^{2} } }}{{\overline{x}}}} \\ \end{array}$$

2 $$\begin{array}{*{20}c} {{\text{RMSSD}}\left( x \right) = \sqrt {\frac{1}{N}\mathop \sum \limits_{i = 1}^{N} (\left| {x_{i - 1} } \right.\left. { - x_{i} } \right|^{2} } )} \\ \end{array}$$

**Quantitative time series analysis:** CV: coefficient of variation; RMSSD: root mean square of successive differences; N: number of samples; $x$: measured variable; $\overline{x}$: mean of $x$.

#### Detrended fluctuation analysis

While quantitative time series analysis is used to measure short- and long-term variations, detrended fluctuation analysis (DFA) is used to detect long-range correlations in time series [10, 11]. We will address detrended fluctuation analysis as “*correlated variability.*” It is based on the assumption that variations are due to extrinsic stimuli that cause local effects or the intrinsic dynamics of the system causing long-range correlations. To quantify the intrinsic variability of the system, the local effects are subtracted. The algorithm consists of several steps, starting with a time series of the measured variable $x$, such as tidal volume or respiratory rate. A new integrated time series $X_{T}$ is calculated by summing the differences between the individual value $x_{i}$ and the average $\overline{x}$ for all values (Eq. 3). Subsequently, this trend function $X_{T}$ is divided into epochs of length $n$, and the local least squares fit (local trend) $Y_{T}$ within this time window is subtracted. The fluctuation $F\left( n \right)$ is then calculated as the root mean square of the integrated and detrended time series (Eq. 4). Finally, this process is repeated over different epochs $n$ and a log–log graph of $F\left( n \right)$ against $n$ is constructed. The slope of a straight line fit yields the parameter *α,* which characterises long-term correlations (Eq. 5). While a higher α-value of 0.5 indicates a time series without any long-term correlations, an increased α-value would suggest the presence of such correlations. One major disadvantage of DFA is that it requires large datasets (i.e. more than 8000 data points) for meaningful interpretations [12].

3 $$\begin{array}{*{20}c} {X\left( t \right) = \mathop \sum \limits_{i = 1}^{t} \left| {x\left( i \right) - \overline{x}} \right|} \\ \end{array}$$

4 $$\begin{array}{*{20}c} {F\left( n \right) = \sqrt {\frac{1}{N}\mathop \sum \limits_{t = 1}^{N} \left[ {X\left( t \right) - Y\left( t \right)} \right]^{2} } } \\ \end{array}$$

5 $$\begin{array}{*{20}c} {F\left( n \right) \propto n^{\alpha } } \\ \end{array}$$

**Detrended fluctuation analysis:** $x\left( t \right)$: measured variable, $\overline{x}$: mean of $x$; $n$: epoch length, $Y\left( t \right)$: local least-squares fit, $N$: number of measurements, $\alpha$: scaling component characterising the extent of long-term correlations.

#### Entropy analysis

Entropy analysis measures the degree of disorder or randomness in the data. In other words, it quantifies the amount of “surprise” or “new information” introduced to an otherwise predictable system. Entropy analysis, therefore, reflects the degree of complexity or “*informational variability.*” This elegant analysis approach is often applied to thermodynamics, but it can also be used to analyse breathing irregularities [13, 14]. Data are considered more irregular and unpredictable when observed patterns are not followed by similar patterns. For this purpose, the entropy analysis algorithm evaluates whether a sequence of data points of length *m* is similar to other sequences in the data within a specified tolerance *r*. Subsequently, the difference between the logarithmic frequencies of similar runs of length m and length *m* + 1 is measured. When the data contain several repetitive patterns, the approximate entropy (ApEn) is low; otherwise, the algorithm yields a higher ApEn. One shortcoming of the calculation of ApEn is its dependency on the choice of sequence length *m* and tolerance *r* because other choices of *m* and *r* can lead to different conclusions on the randomness of the data.

6 $$\begin{array}{*{20}c} {C_{im} \left( r \right) = \frac{{n_{im} \left( r \right)}}{N - m + 1}} \\ \end{array}$$

7 $$\begin{array}{*{20}c} {{\text{ApEn}}\left( {S_{N} ,m,r} \right) = \ln \frac{{C_{m} \left( r \right)}}{{C_{m + 1} \left( r \right)}}} \\ \end{array}$$

**Approximate entropy analysis:** $r$: similarity criterion; $n_{im} \left( r \right)$: number of patterns that are similar within $r$; $m$: pattern length; $S_{N}$: sequence of $N$ measurements; $C_{m} \left( r \right)$: mean of all $C_{im} \left( r \right)$ values; $ApEn$: approximate entropy.

### Conditions and diseases influencing respiratory variability

Breathing variability is modified and influenced by several conditions, such as sleep, cognitive function, age, hypoxia, and diseases (Table 3).

#### Sleep

In healthy participants, quantitative respiratory variability decreases during non-rapid eye movement (non-REM) sleep [15, 16]. In addition, breath-to-breath components display a strong relationship between one breath and another at a time lag of a few breaths (short-term correlations). The regulation of respiratory timing and drive is characterised by additional long-term correlations only during the transition from non-REM to REM sleep [17]. The quantitative variability of respiratory rate is augmented during REM sleep, compared to non-REM sleep, but less prominent than during wakefulness [18]. The decreased quantitative variability of respiration during non-REM sleep is a result of an autoregressive process (i.e. breaths depend linearly on their previous values), as well as a result of periodic oscillations and uncorrelated white noise [19]. The observed breath-to-breath dependence (autoregression) is likely caused by the central respiratory pattern generator, whereas periodic oscillations are more likely to originate from chemical feedback systems [19]. It is unknown whether similar changes can be observed during sedation and anaesthesia with preserved spontaneous respiration.

#### Cognitive load

Changes in cognitive activity also influence respiratory variability [20]. Arithmetic tasks under stressful conditions significantly increased the quantitative variability of respiratory rate, tidal volume, and minute volume by 13%, 50%, and 30%, respectively, compared with measurements during restful watching of the documentary titled “*The March of the Penguins”* [21]. In another mental load experiment, however, quantitative and correlated variability of respiratory rate was reduced by 30% when volunteers were exposed to multiple tasks while assessing their perceptual speed, spatial orientation, and working memory capacity. Breathing variability was restored to its baseline values during the recovery period [22]. The duration and complexity of cognitive load have variable effects on respiratory variability.

#### Exercise

Increasing metabolic demands during physical exercise induces an increase in minute ventilation through the augmentation of the respiratory rate and tidal volume. However, the quantitative variability of the respiratory rate decreases during recumbent bicycle exercise [23]. The correlated variability also decreases, resulting in a more random pattern of breathing, which suggests that the respiratory control system may be operating in a less stable state during exercise [23]. The degree of athletic fitness altered the effect of exercise on breathing variability. In a cardiopulmonary exercise test at maximum oxygen consumption, sedentary volunteers showed a 40% higher quantitative variability of minute ventilation than professional football players from Brazil [24]. In patients with heart failure, a breathing pattern with cyclic fluctuations in minute ventilation during incremental exercise (“exertional oscillatory ventilation”) is a strong predictor of poor prognosis and reflects advanced disease [25].

#### Age

Respiratory variability increases with age. Healthy older adults [60–81 years] showed greater quantitative variability than younger participants [21–50 years], especially during non-REM sleep [16, 26]. Asleep older adults more often show an increase in upper airway resistance, which produces breathing fluctuations caused by mechanical limitations contributing to this breath-to-breath variability of tidal volume [16]. A study of long-term correlations (120 min) shows long-range correlations extending over hundreds of breathing cycles during sleep, and these correlations seem to degrade in older men [11]. The authors suggest that this loss of long-term correlations in breathing dynamics may be caused by intrinsic factors associated with aging such as neuronal dropout, loss of central signal integration, stiffening of the pulmonary parenchyma, and reduced chemoreceptor sensitivity. It is unknown whether these changes in quantitative and correlated variability of breathing are accompanied by changes in complexity or informational variability.

#### Hypoxia and high altitude

Specific changes in respiratory patterns are observed under hypoxic conditions [27, 28]. During the transition from normoxia to isocapnic hypoxia, the quantitative variability of minute ventilation increases by 70%, and that of tidal volume increases by 50%, without inducing periodic breathing [28]. This was mainly mediated by an increase in the random fraction, as the correlated variability decreased [28]. Hypoxic conditions at high altitudes induced similar changes. The incidence of periodic breathing with apneic episodes and their duration also increased as a function of altitude in non-acclimatized participants [29]. Breathing patterns oscillate periodically between clusters of breaths and periods of short apnea during NREM sleep under hypobaric hypoxia [27]. This pattern increases the variability of breathing, quantified in terms of the coefficient of variation. This periodic breathing pattern does not occur during wakefulness or REM sleep and cannot be observed during normocapnia.

#### Arterial hypertension

In addition, arterial hypertension may influence breathing variability. The quantitative variability of tidal volume, respiratory rate, and minute ventilation was significantly higher in a cohort of women at rest with elevated systolic blood pressure [30]. The incidence of apneic events (> 10 s) during resting wakefulness increased more than twofold in patients with elevated blood pressure, whereas the incidence of these breathing pauses did not differ during overnight sleep. It has not been established if periodic breathing is a cause or consequence of long-term hypertension; however, it has been hypothesised that increased sympathetic and/or decreased parasympathetic activity may account for the breathing pattern.

#### Endotoxin

The systemic inflammatory response to endotoxins induces alterations in respiratory frequency and minute ventilation, as well as the respiratory pattern [31]. Even though the overall quantitative variability remained unaltered, the correlated variability of respiratory rate was 42% higher after exposure to endotoxins than placebo [31]. These changes were functions of changes in P_a_CO_2_, suggesting a role of chemical feedback loops. Interestingly, ibuprofen suppressed the increase in the correlated behaviour of respiratory frequency.

#### Anxiety

Marked differences in breathing variability have been reported in children and adolescents with anxiety disorders, such as panic disorder [32, 33]. During resting wakefulness, patients at risk of panic attacks have a twofold higher quantitative variability of tidal volume than healthy controls. This difference persisted even after treatment [34]. In patients with hyperventilation disorder, there is an augmented degree of quantitative variability and a concomitant augmentation in the complexity of tidal volume variability [35]. One study reported increased quantitative and informational respiratory variability in patients with panic disorder only in the standing (but not in the supine) position. The authors hypothesised that this difference may be attributed to a diminished vagal tone in patients with panic disorders [36].

#### Obstructive lung disease

Asthma and chronic obstructive pulmonary disease (COPD) affect breathing variability in different ways. Patients with COPD have higher quantitative variabilities of minute ventilation and tidal volume than age-and sex-matched controls [37]. They have significantly fewer sighs; even after the exclusion of sighs from analysis, respiratory variability was lower in patients with COPD [37]. This reduced variability in breathing patterns may reflect changes in the mechanics of the lung and chest wall or neural adjustments in breathing control.

In contrast, the variability of respiration is increased in patients with asthma, and this effect is related to disease severity. The quantitative variability of tidal breathing parameters in children with asthma aged 7–16 years was 25% greater than that in age-matched children, independent of the use of a bronchodilator [38]. In young patients [3–7 years] with risk factors for asthma, compared to a control group, the quantitative variability of tidal flow is significantly increased [39]. Patients treated with inhalational corticosteroids, compared to the low-risk group, showed a normal tidal flow pattern, suggesting that control medication modifies disease activity and lung function variability.

These distinctively different effects of COPD and asthma on breathing variability were confirmed by a study that used the forced oscillation technique [40] to determine the variability of respiratory impedance and other airway properties. In this study, the temporal dynamics of respiratory impedance was used to distinguish the asthma and COPD groups [41].

#### Restrictive lung disease

Variability in breathing was also reduced in patients with restrictive lung disease. For instance, during resting wakefulness, the quantitative variability of tidal volume was reduced by 56%, inspiratory time was reduced by 33%, expiratory time was reduced by 46%, and minute ventilation was reduced by 43% compared to those of healthy participants [42]. Concurrently, the correlated variability increased, suggesting that patients with restrictive lung disease adopt a constrained breathing pattern.

#### Lung prematurity

Variability in breathing is also influenced by lung maturity. In preterm infants (i.e. born before 37 gestational weeks), quantitative variability of tidal volume and end-tidal expiratory CO_2_ is significantly lower in patients with chronic lung disease of infancy than in patients without supplementary oxygen or CPAP [43]. At the postmenstrual age of 42–50 weeks, the quantitative variability of airway resistance is significantly higher in term infants than in preterm infants [44]. In preterm born infants, lower quantitative variability of tidal volume at a postmenstrual age of 44 weeks is an important predictor of re-hospitalization due to respiratory disease in infancy [45].

### Perioperative period

Various drugs administered during the perioperative period influence breathing control. Propofol decreases the respiratory response to hypoxia and hypercarbia [46, 47] resulting in decreased tidal volume and minute ventilation. Opiates cause dose-dependent hypoventilation mainly through a decrease in respiratory rate [48]. This respiratory depression is often accompanied by increased quantitative tidal volume variability [49]. Midazolam reduces minute ventilation mainly through decreases in tidal volume and, to a lesser extent, respiratory rate [50]. On the other hand, s-ketamine activates breathing with an increase in respiratory rate and inspiratory time and can antagonise opiate-induced hypoventilation [51–55]. The exact effects of these drugs on respiratory variability have not been established. The quantitative variability of respiratory rate was lower than that of tidal volume during the first 24 postoperative hours in patients undergoing major abdominal surgery. These findings suggest that the adaptations of alveolar ventilation to metabolic needs may be predominately achieved by variations in tidal volume [56].

### Intensive care

During weaning from mechanical ventilation, variability in breathing may be valuable for clinical decision-making [57]. Weaning is often preceded by a spontaneous breathing trial (SBT) in which patients are disconnected from ventilatory support to assess the adequacy of their respiratory function. In patients recovering from a systemic inflammatory response syndrome, quantitative variability of tidal volume during a 30 min SBT was significantly lower in patients requiring reinstitution of non-invasive or invasive mechanical ventilation within 48 h [58]. Similar observations were reported in another study of ventilated patients who underwent a 60-min SBT with oxygen supplementation. Patients with a lower quantitative variability of tidal volume during SBT more often require ventilatory support after weaning from mechanical ventilation [59].

Interesting findings were reported in a study of patients requiring prolonged ventilation (> 7 days) [60]. During SBT with CPAP, the quantitative variability of tidal volume, but not respiratory rate, was higher in patients with failed weaning. Concurrently, the informational variability was higher in patients with failed weaning, suggesting a less predictable breathing pattern [61]. An important limitation of this study is that the patients were supported with a mean of 12 ± 4.6 cm H_2_O of pressure support although the aim was to assess the intrinsic variability of a patient [62].

The quantitative variability of the respiratory rate increases significantly during the reduction or interruption of sedation with propofol, midazolam, or their combination [63]. This restoration of respiratory rate variability is greater in patients with lower multiple organ dysfunction scores (MODS) [63].

Various studies on the prognostic value of respiratory variability are presented in Table 4. These findings are interesting; however, we need more data to determine the exact role of variability analyses in guiding clinical decisions.

**Table 4:** Clinical predictive value of respiratory variability

Setting	Reference	Respiratory measurements	Measure of variability	
Preterm infants	Usemann et al. [45]	Full face mask, flowmeter	CV	With versus without later hospitalization due to respiratory disease: TV 0.07 ± 0.03 versus 0.09 ± 0.03* For each IQR increase in CV_VT_, OR for rehospitalization increased by 2.25
ICU weaning trial with 5 cmH_2_O PSV plus 5 cmH_2_O PEEP	Bien et al. [58]	Ventilator	CV	Weaning failure versus weaning success: TV 0.18 ± 0.09 versus 0.28 ± 0.15* BT 0.20 ± 0.12 versus 0.31 ± 0.15* PIF 0.10 ± 0.05 versus 0.16 ± 0.06* AUC: TV 0.75 ± 0.06 PIF 0.80 ± 0.05
ICU spontaneous breathing trial without ventilatory support	Wysocki et al. [59]	Ventilator	CV	Extubation failure versus extubation success: TV 0.18 (0.10–0.24) versus 0.25 (0.14–0.51)* BT 0.14 (0.10–0.21) versus 0.20 (0.13–0.53)*
ICU weaning trial, CPAP 5 cmH_2_O	El Khatib et al. [60]	Ventilator	CV	Extubation failure versus extubation success: TV 0.26 ± 0.07 versus 0.09 ± 0.04* PIF 0.30 ± 0.12 versus 0.12 ± 0.04*
			Kolmogorov entropy	Extubation failure versus extubation success: 0.39 ± 0.09 versus 0.09 ± 0.03*
			Dimension	3.39 ± 0.47 versus 1.33 ± 0.07*
ICU weaning trial with 12 ± 4.6 cmH_2_O PSV plus 5 cmH_2_O PEEP	Engeron [61]	Ventilator	ApEn	Extubation failure versus extubation success: TV 0.328 ± 0.049 versus 0.185 ± 0.027* RR 0.454 ± 0.022 versus 0.459 ± 0.013 AUC: TV 0.74 ± 0.07

CV coefficient of variation, TV tidal volume, IQR interquartile range, OR odds ratio, ICU intensive care unit, PSV pressure support ventilation, PEEP positive end-expiratory pressure, BT breathing time, PIF peak inspiratory flow, AUC area under the curve, ApEn approximate entropy*P < 0.05

### Future research

There is increasing attention for temporal variations of physiologic variables, such as heart rate variability [64], and the amount of available data on breathing variability is increasing. Further research should elucidate the correlation between respiratory variability during the postoperative period and clinically relevant outcomes, such as postoperative morbidity and mortality [65]. Large amounts of physiological data are generated during anaesthesia and intensive care. Technological advances in data analysis, smart learning techniques, and artificial intelligence can facilitate the determination of patients at risk [66–68]. Integrating complex data from multiple sources may lead to improved risk stratification. Recent advances allow us to monitor respiratory function in a continuous and noninvasive manner [50, 56, 69–82]. This is important as postoperative pulmonary complications remain a major disease burden [83–87].

## Conclusions

The variability of respiration over time may be a promising tool for identifying patients at risk of pulmonary complications. The variability of respiration is complex and not fully understood yet. Measuring the variability of a single parameter, such as the respiratory rate, does not necessarily reflect the variability of the respiratory system as a whole. The overall variability of breathing is decreased by COPD, restrictive lung disease, chronic lung disease of infancy, non-REM sleep, and highly demanding cognitive tasks. In contrast, it is increased in older adults during the performance of complex arithmetic tasks during hypoxia and in patients with asthma, hypertension, or anxiety disorder. Further research is required to elucidate the full potential of respiratory variability in critical care and anaesthesiology.

## Data Availability

Not applicable.

## Abbreviations

CV: Coefficient of variation
RMSSD: Root mean square of successive differences
DFA: Detrended fluctuation analysis
ApEn: Approximate entropy
REM: Rapid eye movement
COPD: Chronic obstructive pulmonary disease
CLDI: Chronic lung disease of infancy
CPAP: Continuous positive airway pressure
SBT: Spontaneous breathing trial

## References

[1] Seely AJ, Macklem PT (2004). Complex systems and the technology of variability analysis. Crit Care.

[2] van den Aardweg JG, Karemaker JM (1991). Respiratory variability and associated cardiovascular changes in adults at rest. Clin Physiol.

[3] Priban IP (1963). An analysis of some short-term patterns of breathing in man at rest. J Physiol.

[4] Lewis J, Bachoo M, Polosa C, Glass L (1990). The effects of superior laryngeal nerve stimulation on the respiratory rhythm: phase-resetting and aftereffects. Brain Res.

[5] Bartlett D (1989). Respiratory functions of the larynx. Physiol Rev.

[6] Davis JN, Stagg D (1975). Interrelationships of the volume and time components of individual breaths in resting man. J Physiol.

[7] Benchetrit G, Bertrand F (1975). A short-term memory in the respiratory centres: Statistical analysis. Respir Physiol.

[8] Carley DW, Shannon DC (1988). A minimal mathematical model of human periodic breathing. J Appl Physiol (1985).

[9] Van den Aardweg JG, Karemaker JM (2002). Influence of chemoreflexes on respiratory variability in healthy subjects. Am J Respir Crit Care Med.

[10] Kantelhardt JW (2001). Detecting long-range correlations with detrended fluctuation analysis. Phys A Stat Mech Appl.

[11] Peng CK, Mietus JE, Liu Y, Lee C, Hausdorff JM, Stanley HE (2002). Quantifying fractal dynamics of human respiration: age and gender effects. Ann Biomed Eng.

[12] Peng CK, Havlin S, Stanley HE, Goldberger AL (1995). Quantification of scaling exponents and crossover phenomena in nonstationary heartbeat time series. Chaos.

[13] Pincus SM (1991). Approximate entropy as a measure of system complexity. Proc Natl Acad Sci U S A.

[14] Goldberger AL, Amaral LA, Glass L, Hausdorff JM, Ivanov PC, Mark RG (2000). PhysioBank, PhysioToolkit, and PhysioNet: components of a new research resource for complex physiologic signals. Circulation.

[15] Chapman KR, Bruce EN, Gothe B, Cherniack NS (1988). Possible mechanisms of periodic breathing during sleep. J Appl Physiol (1985).

[16] Hudgel DW, Devadatta P, Hamilton H (1993). Pattern of breathing and upper airway mechanics during wakefulness and sleep in healthy elderly humans. J Appl Physiol (1985).

[17] Rostig S, Kantelhardt JW, Penzel T, Cassel W, Peter JH, Vogelmeier C (2005). Nonrandom variability of respiration during sleep in healthy humans. Sleep.

[18] Gutierrez G, Williams J, Alrehaili GA, McLean A, Pirouz R, Amdur R (2016). Respiratory rate variability in sleeping adults without obstructive sleep apnea. Physiol Rep.

[19] Modarreszadeh M, Bruce EN, Gothe B (1990). Nonrandom variability in respiratory cycle parameters of humans during stage 2 sleep. J Appl Physiol (1985).

[20] Grassmann M, Vlemincx E, von Leupoldt A, Mittelstadt JM, Van den Bergh O (2016). Respiratory changes in response to cognitive load: a systematic review. Neural Plast.

[21] Vlemincx E, Taelman J, De Peuter S, Van Diest I, Van den Bergh O (2011). Sigh rate and respiratory variability during mental load and sustained attention. Psychophysiology.

[22] Grassmann M, Vlemincx E, von Leupoldt A, Van den Bergh O (2016). The role of respiratory measures to assess mental load in pilot selection. Ergonomics.

[23] Busha BF (2010). Exercise modulation of cardiorespiratory variability in humans. Respir Physiol Neurobiol.

[24] Castro RRT, Lima SP, Sales ARK, Nobrega A (2017). Minute-ventilation variability during cardiopulmonary exercise test is higher in sedentary men than in Athletes. Arq Bras Cardiol.

[25] Corra U, Giordano A, Bosimini E, Mezzani A, Piepoli M, Coats AJ (2002). Oscillatory ventilation during exercise in patients with chronic heart failure: clinical correlates and prognostic implications. Chest.

[26] Tobin MJ, Mador MJ, Guenther SM, Lodato RF, Sackner MA (1988). Variability of resting respiratory drive and timing in healthy subjects. J Appl Physiol (1985).

[27] Berssenbrugge A, Dempsey J, Iber C, Skatrud J, Wilson P (1983). Mechanisms of hypoxia-induced periodic breathing during sleep in humans. J Physiol.

[28] Jubran A, Tobin MJ (2000). Effect of isocapnic hypoxia on variational activity of breathing. Am J Respir Crit Care Med.

[29] Waggener TB, Brusil PJ, Kronauer RE, Gabel RA, Inbar GF (1984). Strength and cycle time of high-altitude ventilatory patterns in unacclimatized humans. J Appl Physiol Respir Environ Exerc Physiol.

[30] Anderson DE, McNeely JD, Chesney MA, Windham BG (2008). Breathing variability at rest is positively associated with 24-h blood pressure level. Am J Hypertens.

[31] Preas HL, Jubran A, Vandivier RW, Reda D, Godin PJ, Banks SM (2001). Effect of endotoxin on ventilation and breath variability: role of cyclooxygenase pathway. Am J Respir Crit Care Med.

[32] Pine DS, Coplan JD, Papp LA, Klein RG, Martinez JM, Kovalenko P (1998). Ventilatory physiology of children and adolescents with anxiety disorders. Arch Gen Psychiatry.

[33] Grassi M, Caldirola D, Vanni G, Guerriero G, Piccinni M, Valchera A (2013). Baseline respiratory parameters in panic disorder: a meta-analysis. J Affect Disord.

[34] Martinez JM, Kent JM, Coplan JD, Browne ST, Papp LA, Sullivan GM (2001). Respiratory variability in panic disorder. Depress Anxiety.

[35] Bokov P, Fiamma MN, Chevalier-Bidaud B, Chenivesse C, Straus C, Similowski T (2016). Increased ventilatory variability and complexity in patients with hyperventilation disorder. J Appl Physiol (1985).

[36] Yeragani VK, Radhakrishna RK, Tancer M, Uhde T (2002). Nonlinear measures of respiration: respiratory irregularity and increased chaos of respiration in patients with panic disorder. Neuropsychobiology.

[37] Loveridge B, West P, Anthonisen NR, Kryger MH (1984). Breathing patterns in patients with chronic obstructive pulmonary disease. Am Rev Respir Dis.

[38] Hmeidi H, Motamedi-Fakhr S, Chadwick E, Gilchrist FJ, Lenney W, Iles R (2017). Tidal breathing parameters measured using structured light plethysmography in healthy children and those with asthma before and after bronchodilator. Physiol Rep.

[39] Seppa VP, Pelkonen AS, Kotaniemi-Syrjanen A, Viik J, Makela MJ, Malmberg LP (2016). Tidal flow variability measured by impedance pneumography relates to childhood asthma risk. Eur Respir J.

[40] Que CL, Kenyon CM, Olivenstein R, Macklem PT, Maksym GN (2001). Homeokinesis and short-term variability of human airway caliber. J Appl Physiol (1985).

[41] Muskulus M, Slats AM, Sterk PJ, Verduyn-Lunel S (2010). Fluctuations and determinism of respiratory impedance in asthma and chronic obstructive pulmonary disease. J Appl Physiol (1985).

[42] Brack T, Jubran A, Tobin MJ (2002). Dyspnea and decreased variability of breathing in patients with restrictive lung disease. Am J Respir Crit Care Med.

[43] Fouzas S, Theodorakopoulos I, Delgado-Eckert E, Latzin P, Frey U (2017). Breath-to-breath variability of exhaled CO2 as a marker of lung dysmaturity in infancy. J Appl Physiol (1985).

[44] Usemann J, Demann D, Anagnostopoulou P, Korten I, Gorlanova O, Schulzke S (2017). Interrupter technique in infancy: Higher airway resistance and lower short-term variability in preterm versus term infants. Pediatr Pulmonol.

[45] Usemann J, Suter A, Zannin E, Proietti E, Fouzas S, Schulzke S, et al. Variability of tidal breathing parameters in preterm infants and associations with respiratory morbidity during infancy: a Cohort Study. J Pediatr. 2019;205:61–9 e1. 10.1016/j.jpeds.2018.10.002 30416016

[46] Blouin RT, Seifert HA, Babenco HD, Conard PF, Gross JB (1993). Propofol depresses the hypoxic ventilatory response during conscious sedation and isohypercapnia. Anesthesiology.

[47] Goodman NW, Black AM, Carter JA (1987). Some ventilatory effects of propofol as sole anaesthetic agent. Br J Anaesth.

[48] Pattinson KT (2008). Opioids and the control of respiration. Br J Anaesth.

[49] Bouillon T, Bruhn J, Roepcke H, Hoeft A (2003). Opioid-induced respiratory depression is associated with increased tidal volume variability. Eur J Anaesthesiol EJA.

[50] Gonzalez Castro LN, Mehta JH, Brayanov JB, Mullen GJ (2017). Quantification of respiratory depression during pre-operative administration of midazolam using a non-invasive respiratory volume monitor. PLoS ONE.

[51] Mortero RF, Clark LD, Tolan MM, Metz RJ, Tsueda K, Sheppard RA (2001). The effects of small-dose ketamine on propofol sedation: respiration, postoperative mood, perception, cognition, and pain. Anesth Analg.

[52] Eikermann M, Grosse-Sundrup M, Zaremba S, Henry ME, Bittner EA, Hoffmann U (2012). Ketamine activates breathing and abolishes the coupling between loss of consciousness and upper airway dilator muscle dysfunction. Anesthesiology.

[53] Oliveira GS (2014). The effect of ketamine on hypoventilation during deep sedation with midazolam and propofol: a randomised, double-blind, placebo-controlled trial. Eur J Anaesthesiol.

[54] Persson J, Scheinin H, Hellstrom G, Bjorkman S, Gotharson E, Gustafsson LL (1999). Ketamine antagonises alfentanil-induced hypoventilation in healthy male volunteers. Acta Anaesthesiol Scand.

[55] Mildh L, Taittonen M, Leino K, Kirvela O (1998). The effect of low-dose ketamine on fentanyl-induced respiratory depression. Anaesthesia.

[56] van den Bosch OFC, Alvarez-Jimenez R, Stam MMH, den Boer FC, Loer SA. Variations in respiratory rate do not reflect changes in tidal volume or minute ventilation after major abdominal surgery. J Clin Monit Comput. 2021;35(4):787–96. 10.1007/s10877-020-00538-3 PMC8286957 32488678

[57] Seely AJ, Bravi A, Herry C, Green G, Longtin A, Ramsay T (2014). Do heart and respiratory rate variability improve prediction of extubation outcomes in critically ill patients?. Crit Care.

[58] Bien MY, Hseu SS, Yien HW, Kuo BI, Lin YT, Wang JH (2004). Breathing pattern variability: a weaning predictor in postoperative patients recovering from systemic inflammatory response syndrome. Intensive Care Med.

[59] Wysocki M, Cracco C, Teixeira A, Mercat A, Diehl JL, Lefort Y (2006). Reduced breathing variability as a predictor of unsuccessful patient separation from mechanical ventilation. Crit Care Med.

[60] El-Khatib M, Jamaleddine G, Soubra R, Muallem M (2001). Pattern of spontaneous breathing: potential marker for weaning outcome. Spontaneous breathing pattern and weaning from mechanical ventilation. Intensive Care Med.

[61] Engoren M (1998). Approximate entropy of respiratory rate and tidal volume during weaning from mechanical ventilation. Crit Care Med.

[62] Brochard L (1998). Breathing: does regular mean normal?. Crit Care Med.

[63] Bradley BD, Green G, Ramsay T, Seely AJ (2013). Impact of sedation and organ failure on continuous heart and respiratory rate variability monitoring in critically ill patients: a pilot study. Crit Care Med.

[64] Buchman TG, Stein PK, Goldstein B (2002). Heart rate variability in critical illness and critical care. Curr Opin Crit Care.

[65] van de Borne P (2004). Variability science in intensive care - how relevant is it?. Crit Care.

[66] Hashimoto DA, Witkowski E, Gao L, Meireles O, Rosman G (2020). Artificial intelligence in anesthesiology: current techniques, clinical applications, and limitations. Anesthesiology.

[67] Lovejoy CA, Buch V, Maruthappu M (2019). Artificial intelligence in the intensive care unit. Crit Care.

[68] Hofer IS, Burns M, Kendale S, Wanderer JP (2020). Realistically integrating machine learning into clinical practice: a road map of opportunities, challenges, and a potential future. Anesth Analg.

[69] van Loon K, van Zaane B, Bosch EJ, Kalkman CJ, Peelen LM (2015). Non-invasive continuous respiratory monitoring on general hospital wards: a systematic review. PLoS ONE.

[70] Cavalcante AN, Martin YN, Sprung J, Imsirovic J, Weingarten TN (2018). Low minute ventilation episodes during anesthesia recovery following intraperitoneal surgery as detected by a non-invasive respiratory volume monitor. J Clin Monit Comput.

[71] Ebert TJ, Middleton AH, Makhija N (2017). Ventilation monitoring during moderate sedation in GI patients. J Clin Monit Comput.

[72] Holley K, MacNabb CM, Georgiadis P, Minasyan H, Shukla A, Mathews D (2016). Monitoring minute ventilation versus respiratory rate to measure the adequacy of ventilation in patients undergoing upper endoscopic procedures. J Clin Monit Comput.

[73] Ianchulev S, Ladd D, MacNabb CM, Qin L, Marengi N, Freeman J (2017). Use of a respiratory volume monitor to assess respiratory competence in cardiac surgery patients after extubation. J Clin Med Res.

[74] Kodali BS, Choi L, Chau A, Harvey BC, Brayanov J, Tsen LC (2020). Use of a novel non-invasive respiratory monitor to study changes in pulmonary ventilation during labor epidural analgesia. J Clin Monit Comput.

[75] Mathews DM, Oberding MJ, Simmons EL, O'Donnell SE, Abnet KR, MacDonald K (2018). Improving patient safety during procedural sedation via respiratory volume monitoring: a randomized controlled trial. J Clin Anesth.

[76] Mehta JH, Cattano D, Brayanov JB, George EE (2017). Assessment of perioperative minute ventilation in obese versus non-obese patients with a non-invasive respiratory volume monitor. BMC Anesthesiol.

[77] Nichols RH, Blinn JA, Ho TM, McQuitty RA, Kinsky MP (2018). Respiratory volume monitoring reduces hypoventilation and apnea in subjects undergoing procedural sedation. Respir Care.

[78] Schumann R, Harvey B, Zahedi F, Bonney I (2019). Minute ventilation assessment in the PACU is useful to predict postoperative respiratory depression following discharge to the floor: a prospective cohort study. J Clin Anesth.

[79] Schumann R, Kwater AP, Bonney I, Ladd D, Kim J, Gupta A (2016). Respiratory volume monitoring in an obese surgical population and the prediction of postoperative respiratory depression by the STOP-bang OSA risk score. J Clin Anesth.

[80] Voscopoulos C, Brayanov J, Ladd D, Lalli M, Panasyuk A, Freeman J (2013). Special article: evaluation of a novel noninvasive respiration monitor providing continuous measurement of minute ventilation in ambulatory subjects in a variety of clinical scenarios. Anesth Analg.

[81] Williams GW, George CA, Harvey BC, Freeman JE (2017). A comparison of measurements of change in respiratory status in spontaneously breathing volunteers by the ExSpiron noninvasive respiratory volume monitor versus the Capnostream capnometer. Anesth Analg.

[82] Zhang X, Kassem MA, Zhou Y, Shabsigh M, Wang Q, Xu X (2017). A brief review of non-invasive monitoring of respiratory condition for extubated patients with or at risk for obstructive sleep apnea after surgery. Front Med (Lausanne).

[83] Miskovic A, Lumb AB (2017). Postoperative pulmonary complications. Br J Anaesth.

[84] Smetana GW, Lawrence VA, Cornell JE (2006). American College of P. Preoperative pulmonary risk stratification for noncardiothoracic surgery: systematic review for the American College of Physicians. Ann Intern Med.

[85] Smith PR, Baig MA, Brito V, Bader F, Bergman MI, Alfonso A (2010). Postoperative pulmonary complications after laparotomy. Respiration.

[86] Yang CK, Teng A, Lee DY, Rose K (2015). Pulmonary complications after major abdominal surgery: National Surgical Quality Improvement Program analysis. J Surg Res.

[87] Zikria BA, Sencer JL, Kinney JM, Broell JR (1974). Alterations in ventilatory function and breathing patterns following surgical trauma. Ann Surg.

